# Controlling Growth and Osteogenic Differentiation of Osteoblasts on Microgrooved Polystyrene Surfaces

**DOI:** 10.1371/journal.pone.0161466

**Published:** 2016-08-29

**Authors:** Lanying Sun, Daniel Pereira, Qibao Wang, David Baião Barata, Roman Truckenmüller, Zhaoyuan Li, Xin Xu, Pamela Habibovic

**Affiliations:** 1 Shandong Provincial Key Laboratory of Oral Tissue Regeneration, School of Stomatology, Shandong University, Jinan, Shandong Province, China; 2 Oral Implantology Center, Stomatology Hospital of Jinan, Jinan, Shandong Province, China; 3 Department of Tissue Regeneration, MIRA Institute for Biomedical Technology and Technical Medicine, University of Twente, Enschede, Overijssel, The Netherlands; 4 MERLN Institute for Technology-Inspired Regenerative Medicine, Maastricht University, Maastricht, Limburg, The Netherlands; University of California, San Diego, UNITED STATES

## Abstract

Surface topography is increasingly being recognized as an important factor to control the response of cells and tissues to biomaterials. In the current study, the aim was to obtain deeper understanding of the effect of microgrooves on shape and orientation of osteoblast-like cells and to relate this effect to their proliferation and osteogenic differentiation. To this end, two microgrooved polystyrene (PS) substrates, differing in the width of the grooves (about 2 μm and 4 μm) and distance between individual grooves (about 6 μm and 11 μm, respectively) were fabricated using a combination of photolithography and hot embossing. MG-63 human osteosarcoma cells were cultured on these microgrooved surfaces, with unpatterned hot-embossed PS substrate as a control. Scanning electron- and fluorescence microscopy analyses showed that on patterned surfaces, the cells aligned along the microgrooves. The cells cultured on 4 μm-grooves / 11 μm-ridges surface showed a more pronounced alignment and a somewhat smaller cell area and cell perimeter as compared to cells cultured on surface with 2 μm-grooves / 6 μm-ridges or unpatterned PS. PrestoBlue analysis and quantification of DNA amounts suggested that microgrooves used in this experiment did not have a strong effect on cell metabolic activity or proliferation. However, cell differentiation towards the osteogenic lineage was significantly enhanced when MG-63 cells were cultured on the 2/6 substrate, as compared to the 4/11 substrate or unpatterned PS. This effect on osteogenic differentiation may be related to differences in cell spreading between the substrates.

## Introduction

Establishing successful integration of a biomedical implant into the host bone tissue is of prime importance in orthopedics and dental surgery [1–4]. Efforts invested in optimizing the interface between an implant and its biological environment are growing, as a result of a widespread use of, for example, dental implants. Surface-structural features of biomaterials in the form of roughness and topography, are, in addition to surface-chemical properties, increasingly being recognized as crucial factor to control the response of cells and tissues to biomaterials [5–10]. Surface topography has been shown important for the early events of attachment and formation of focal adhesions, activating mechanotransduction events, which eventually may be determinant for cell fate and consequent tissue formation.

Among various types of designed topographies, microsized grooved surfaces have been extensively studied for their effects on cell alignment because they can be relatively easily produced using a variety of microfabrication techniques [4, 8, 11–16]. Regarding the behavior of osteogenic cells on grooved surfaces, it has been demonstrated that *in vitro*, they strongly orient in the direction of grooves, unlike on flat surfaces, where a random orientation is generally observed [5, 16–19]. It has also been demonstrated that microgrooves with widths comparable to the cell size induce remarkable cell guidance, while the effect of the grooves with widths appreciably larger than the cells is weak [20]. In a study of SaOs-2 osteoblastic cells on microgrooves with widths ranging from 4 to 38 μm, it was shown that the narrower grooves (4 to 16 μm), in the range of 0.5–2 fold of cell size, are more effective in guiding the cell orientation [17]. A recent report showed that microgrooves with a width ranging from 2 to 12 μm exhibits great contact guidance effects on the shape and orientation of rat bone marrow cells and fibroblasts [14, 21]. Another study showed that microgrooves with a width between 1 and 10 μm can change rat dermal fibroblasts cell morphology and induce cell guidance [11, 22]. In again another study, it was revealed that the degree of cell guidance and alignment is greatest on narrow grooves (2 and 4 μm), a width that is below the MG-63 cell size [16]. However, it was also demonstrated that microgrooves with the width considerably smaller than the cell size, have a less pronounced effects on cell shape. For example, contact guidance was not observed when fibroblast cells were cultured on grooves smaller than 100 nm [23].

While contact guidance is a generally accepted effect of microgrooved surfaces on cell orientation and morphology, there is less consistency in results regarding the effect of such micropatterns on osteoblast proliferation and osteogenic differentiation [12, 13, 24–27]. For example, in the study by Matsuzaka et al. [12], no significant difference in the amount of mineralized extracellular matrix, or alkaline phosphatase (ALP) expression of rat bone marrow cells cultured on polystyrene (PS) were observed as a result of groove depth or width, whereas when the cells were cultured on poly(lactic acid) substrates with a depth of 1 μm and a width of 1 μm or 2 μm, more mineralized extracellular matrix formation was observed than on grooves with larger sizes. Further study by the same group [13] showed no effect on proliferation of rat bone marrow cells as a result of presence of microgrooves on either PS or poly(lactic acid). A higher calcium content was observed on microgrooved poly(lactic acid) as compared to PS, without a significant effect of the microgroove dimensions. Another study, by Kenar et al. [24] showed a positive effect of a substrate made of a blend of poly(3-hydroxybutyrate-*co*-3-hydroxyvalerate) (PHBV) and poly(l/d,l-lactic acid) (P(l/dl)LA) with 27 μm wide grooves, on ALP expression and calcium deposition by rat bone marrow derived osteoblasts, as compared to unpatterned controls. A higher ALP expression by SaOs-2 cells was observed on microgrooved calcium phosphate substrate as compared to flat silicon substrate or tissue culture plastic, although no direct comparison with unpatterned calcium phosphate was made. There was no effect on cell proliferation of micropatterns [25]. Yang et al. [26] showed a significantly higher proliferation of human fetal osteoblasts on microgrooved calcium phosphate ceramic as compared to the unpatterned one, but no consistent results regarding the effect of the groove dimensions were observed. Finally, in the study by Jiang et al. [27], a negative effect of microgrooved titania was observed on both proliferation and ALP expression of MC3T3-E1 cells, as compared to the unpatterned substrate, whereas no differences were observed between 12- or 40 μm wide grooves.

In the current study, the focus was on the properties of microgrooved surfaces, i.e. their size and periodicity. To investigate these properties, hot embossing based on photolithographically patterned micromoulds was used as a means to provide surfaces of PS, with distinct microscale features. As one of the first microfabrication techniques applied to the field of biology and biomedicine, photolithography has been widely used to generate microstructures such as grooves and wells in inorganic materials such as silicon and silicon oxide [28, 29]. This enabled the creation of a more controlled microenvironment and further study of the influence of surface topography on cell behaviour [30, 31].

PS was selected for this study as it is a widely used cell culture substrate, that itself does not promote or inhibit (pre)-osteoblast differentiation towards the osteogenic lineage. Furthermore, this thermoplastic polymer is amenable for precise patterning on the micron- or submicron scale [15], allowing detailed studies into the effect of surface topographies on cell behaviour.

Here, two types of microgrooved PS substrates, differing in the width of the grooves (2 or 4 μm) and the distance between adjacent grooves (6 or 11 μm, respectively) were used, but both having subcellular dimensions to profit from the previously demonstrated contact guidance effect. MG-63 human osteosarcoma cells were cultured on these surfaces, and their attachment, metabolic activity, proliferation and osteogenic differentiation were assessed.

## Materials and Methods

### Micropatterning of PS surfaces

Standard photolithography followed by reactive ion etching was used to produce a silicon wafer carrying two types of periodical patterns of parallel, straight microgrooves. Upon production, the dimensions of topographical features were measured using a white light interferometer (ContourGT-I, Bruker). The patterns were then embossed into PS sheets with a thickness of 50 μm (Goodfellow Ltd.) by applying a pressure of 50 bar at 120°C for 2 min in a nano imprint lithography system (EITRE^®^ 6, Obducat). A PS substrate that was hot-embossed with a flat silicon wafer served as a control. The accuracy of pattern transfer was evaluated by an environmental scanning electron microscope (SEM; XL30, ESEM-FEG, Philips) in the secondary electron mode and quantified again using white light interferometry. For cell culture, substrates with a diameter of 10 mm were punched from the patterned and flat surfaces, followed by activation by air plasma in a plasma cleaner (PDC-002, Harrick Scientific) for 15 seconds. Prior to cell seeding, patterned and flat substrates were placed in ultra-low attachment 48-well-plates, fixed with o-rings and sterilized with 70% ethanol for 15 min with refreshments every 5 min. After complete ethanol evaporation at RT, the samples were washed twice with sterile phosphate buffered saline (PBS) and incubated in cell culture medium overnight.

### Cell culture on patterned PS

Osteoblast-like MG-63 cells (ATCC® CRL-1427^TM^), originally derived from osteosarcoma-affected bone, were maintained in proliferation medium comprising α-minimal essential medium (α-MEM; Gibco), 10% fetal bovine serum (Lonza), 100 U ml^-1^ penicillin (Gibco), 100 μg ml^-1^ streptomycin (Gibco), 0.2 mM ascorbic acid (Sigma-Aldrich), 2 mM L-glutamine (Gibco), and 1 ng ml^-1^ basic fibroblast growth factor (bFGF; Instruchemie). Cells were expanded at 37°C in a humidified atmosphere with 5% CO_2_. Medium was refreshed twice a week. Upon reaching 80% confluence, cells were trypsinized and seeded on the PS substrates, and incubated in 1 mL medium at 37°C in a humidified atmosphere with 5% CO_2_. For cell attachment and morphology analysis, 2500 cells were seeded on each sample in basic cell culture medium (BM; proliferation medium without bFGF). For the assessment of metabolic activity, proliferation and osteogenic differentiation, 4000 MG-63 cells were seeded on each sample and incubated in either BM or osteogenic medium (OM; BM supplemented with 10 nM dexamethasone). Both media were refreshed every 2 to 3 days.

### SEM, fluorescence microscopy and CellProfiler analysis

To assess cell shape on different substrates by SEM and fluorescence microscopy, the cells were cultured for 24 hours in BM. This time point, at which a sufficient number of cells was attached on the surface to allow analysis, but the cells were not confluent yet, was selected based on the previous work, which showed that the effect of surface microfeatures on cell shape occurred early, and the maintenance of this effect was dependent on the properties of the substrate [32, 33].

After 24-hour culture in BM, the cells were washed with PBS, and fixed with 10% formalin (Sigma) for 30 min. For fluorescence microscopy analysis, the samples were permeabilized with 0.1% Triton-X 100 for 5 min and blocked with 1% bovine serum albumin (BSA) in PBS. To stain cell cytoskeleton, Alexa Fluor 488 (1:60 dilution in 1% BSA in PBS; Invitrogen) was added to the samples and incubated for 45 min. Then, DAPI (1:100 dilution in 1% BSA in PBS; Sigma-Aldrich/Fluka) was added for 20 min to stain cell nuclei. Cell images were acquired using an automated fluorescence microscope (BD Pathway™ 435; BD Biosciences) and then analyzed using the CellProfiler software with built-in modules (Measure Object Area Shape) [34]. 6 different areas of each sample and at least 228 cells were used to determine cell area and perimeter as output parameters to compare the effect of different microgrooved topographies.

Samples intended for SEM analysis were rinsed with PBS, dehydrated with a graded series of isopropanol (70, 80, 90, 96 and 100%) for 20–30 min each, and finally completed in hexamethyldisilazane (HMDS; Sigma-Aldrich) 2 times for 15 min. The samples were then air-dried, mounted onto SEM stubs, and sputter-coated with gold.

The orientation angle (OA), which is defined as the angle between the long axis (maximum length) of the cell and the direction of the groove [35], was used to evaluate the contact guidance generated by the microgrooves. A cell that perfectly aligns with the groove should has an OA of 0°, representing the strongest contact guidance introduced by a microgrooved substrate, whereas the OA of a randomly spread cell is close to 45°. OA was calculated for about 40 cells for each topography, based on the SEM images taken from 6 to 8 randomly selected areas.

### Metabolic activity, cell proliferation and ALP activity assay

PrestoBlue^®^, a non-destructive cell viability assay (Life Technologies) containing a growth indicator which is reduced by metabolically active cells to a fluorescent agent, was used to quantitatively analyse cell viability (n = 3) after 7, 14 and 21 days of culture, according to the manufacturer’s protocol. In brief, 1 mL of cell culture medium containing 100 μL of the PrestoBlue reagent was added into each well after washing with PBS. The plate was then incubated at 37°C in a humidified atmosphere with 5% CO_2_ for 40 min. Fluorescence was measured at 590 nm in a spectrophotometer (Victor 3, Perkin Elmer), after which the culture was continued.

To assess proliferation, cells (n = 3) were harvested at 7, 14 and 21 days. Total DNA amount was quantified with CyQuant Cell Proliferation Assay kit (Sigma) as a measure of total cell number, according to the manufacturer’s protocol, and a fluorescence measurement (excitation at 480 nm and emission at 520 nm) using a spectrophotometer (Victor 3, Perkin Elmer).

ALP activity was then determined with a CDP-star assay kit (Roche Applied Science), according to the manufacturer’s instructions. The ALP activity was evaluated by measurement of luminescence using a spectrophotometer (Victor 3, Perkin Elmer) and normalized for the DNA content.

### Osteogenic gene expression by quantitative real-time PCR

To evaluate the effect of microgrooves on the expression of a set of osteogenic genes, MG-63 cells (n = 3) were cultured on samples for 7, 14 and 21 days. Total RNA was extracted by using a combination of TRIzol^®^ (Invitrogen) and a Nucleospin^®^ RNA isolation and purification kit (Macherey-Nagel). In brief, 1 mL of TRIzol reagent was added to each well, followed by one freeze/thaw cycle. After mixing with 200 μL chloroform, the samples were centrifuged and the aqueous phase was collected. 350 μL 70% ethanol was added to each sample before loading onto the RNA binding column of the NucleoSpin RNA II isolation kit. Subsequent steps were in accordance with the manufacturer’s instruction. After quantification using a NanoDrop spectrophotometer (Nanodrop Technologies), RNA samples were reverse-transcribed into cDNA with an iScriptcDNA Synthesis kit (BioRad) according to the manufacturer’s instructions. Quantitative real-time PCR was performed for analyzing expression of bone morphogenetic protein-2 (BMP-2), runt-related transcription factor 2 (Runx2), ALP, collagen Type 1 (Col-1) and osteocalcin (OC). The CT values were normalized to the glyceraldehyde 3-phosphate dehydrogenase (GAPDH) housekeeping gene and fold induction was calculated using the comparative ΔCT method. Primer sequences of the selected markers are listed in Table 1.

**Table 1:** Primer sequences of the osteogenic genes, the expression of which was investigated using qPCR analysis.

Gene	Forward Primer	Reverse Primer
GAPDH	CGCTCTCTGCTCCTCCTGTT	CCATGGTGTCTGAGCGATGT
BMP-2	CCAAGTAAGTCCAACGAAAG	GGTGATGTCCTCGTCTGTA
Runx2	ATGGCGGGTAACGATGAAAAT	ACGGCGGGGAAGACTGTGC
COL-I	AGGGCCAAGACGAAGACATC	AGATCACGTCATCGCACAACA
ALP	ACAAGCACTCCCACTTCATC	TTCAGCTCGTACTGCATGTC
OCN	TGAGAGCCTCACACTCCTC	CGCCTGGGTCTCTTCACTAC

### Statistical analysis

For cell area, perimeter and OA analysis, one-way ANOVA with Bonferroni post-hoc test was used to evaluate the differences between samples. To analyze the topography effect on cell proliferation, metabolic activity, ALP activity and gene expression, two-way ANOVA with Bonferroni post-hoc tests was used. The significance level was set at *p* < 0.05.

## Results

### Characterization of micropatterns

**Fig 1:**
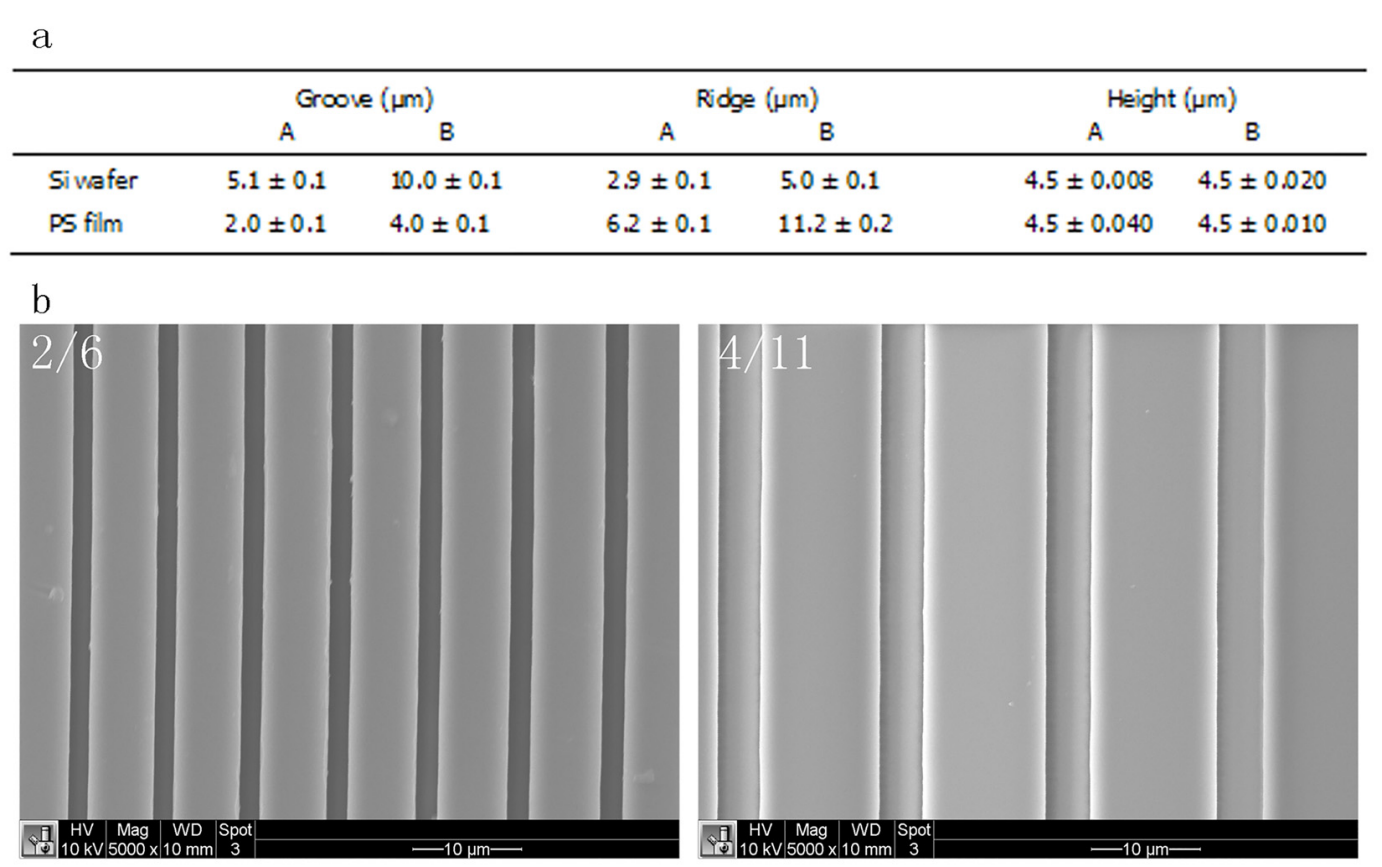
**Dimensions of grooves and ridges of silicon wafers and of respective hot-embossed polystyrene films of the narrow (A, 2/6) and wide (B, 4/11) designs measured using white light interferometry (n = 10) (a) and SEM images of 2/6 and 4/11 (scale bar = 10 μm) (b).** PS films were successfully hot-embossed using the Si wafer. The width of the grooves (ridges on PS substrate) consistently increased with about 1 μm upon hot embossing.

Light interferometry measurements showed that the two patterns of the silicon wafer, used to hot-emboss PS, were different in the width of the grooves and the ridge width, i.e. distance between the grooves (Fig 1A). Pattern A had a groove width of 5.1±0.1 μm and a ridge width of 2.9±0.1, whereas the groove and the ridge width of pattern B were 10.0±0.1 μm and 5.0±0.1 μm, respectively. In both cases, the grooves had the same depth of 4.5 μm. Microgrooved surfaces were successfully hot-embossed on PS substrates, resulting in substrates with groove/ridge width of 2.0±0.1/6.2±0.1 μm (substrate 2/6) and 4.0±0.1/11.2±0.2 μm, (substrate 4/11), respectively (Fig 1).

### Cell attachment, morphology and orientation on micropatterned PS

**Fig 2:**
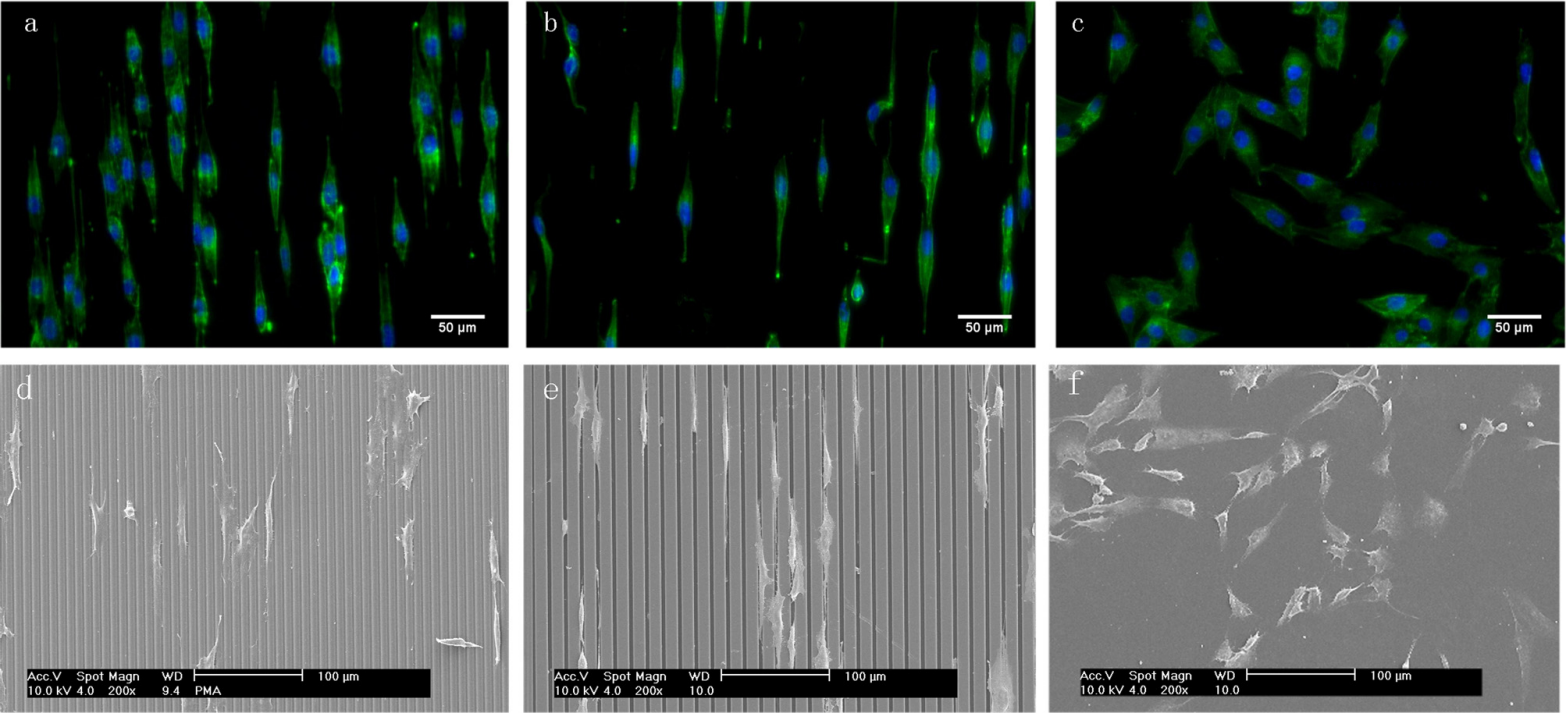
**Fluorescent images of DAPI/phalloidin-stained MG-63 cells (a-c) and SEM images (d–f) after 24-hour attachment on 2/6 (a, d), 4/11 (b, e) and flat control (c, f).** Both microgrooved surfaces induced alignment of the cells in the direction of the grooves. While cells on 2/6 were predominantly found on the ridges, bridging over two or more grooves, on 4/11, the cells were predominantly found inside the grooves. Cells on the flat control appeared randomly oriented and spread.

To investigate the effect of microgrooved topographies on cell attachment and morphology, fluorescence microscopy (Fig 2A–2C) and SEM (Fig 2D–2F) analyses were performed after 24-hour attachment, showing that all surfaces allowed cell attachment and that the cell morphology was dependent on the surface-topographical features. While on the flat, unpatterned PS surface, MG-63 cells were randomly orientated and displayed a spread phenotype with distinct cytoplasmic processes, on the microgrooved surfaces, the cells were aligned in the direction parallel to the grooves with clear elongation of the cytoskeleton. On 2/6, the substrate with narrower grooves and ridges, the cells were predominantly observed on the ridges. A “bridging” effect was occasionally observed, whereby a cell spread over grooves connecting two or more ridges. The cells grown on 4/11, with broader grooves and ridges, appeared more confined to the topographical features. They were predominantly found inside the grooves and on the edges of the ridges, but rarely on top of the ridges. The “groove-bridging” effect was less frequently observed on 4/11 as compared to 2/6.

**Fig 3:**
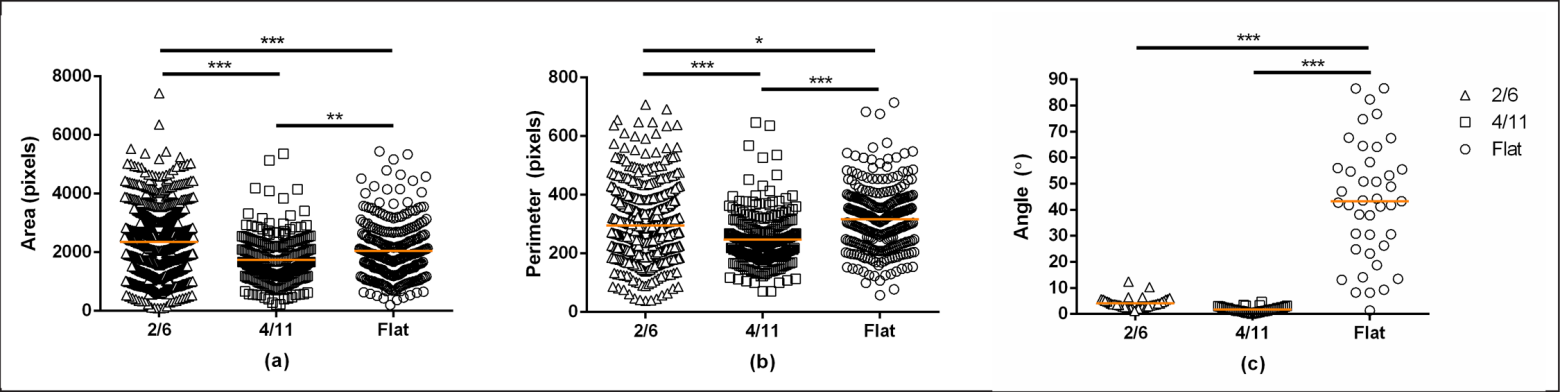
**Quantification of cell area (a), cell perimeter (b) and orientation angle (c) of MG-63 cells cultured for 24 hours on 2/6, 4/11 and flat control.** Cells cultured on 2/6 showed a significantly larger cell area and cell perimeter as compared to cells cultured on 4/11 or the flat control. Furthermore, cells cultured on 4/11 showed a smaller cell area and cell perimeter as compared to the flat control. Both microgroove topographies strongly enhanced cell orientation as compared to cells cultured on the unpatterned PS. Statistically significant differences are marked with * for *p* < 0.05, ** for p < 0.01 and ***for *p* < 0.001.

The CellProfiler analysis of the parameters cell area and cell perimeter (Fig 3A and 3B) confirmed these qualitative observations. Values for both cell area and cell perimeter were higher when cells were cultured on 2/6 than on 4/11 or the flat control. Cells cultured on the unpatterned substrate showed a larger cell area and cell perimeter as compared to the cells cultured on 4/11. These data confirmed the effect of surface topography on cell morphology.

The OA analysis (Fig 3C) was performed after the initial cell attachment to investigate the contact guidance effect of the microgrooves. The cells cultured on the flat substrate had an average OA of 43.29°±25.61°, confirming a random orientation. In contrast, the average OA of the cells seeded on 2/6 and 4/11 was 3.91°±1.03° and 1.91°±0.59°, respectively, suggesting a strong effect of the microgrooves on cell orientation.

### Cell viability and proliferation on micropatterned PS

**Fig 4:**
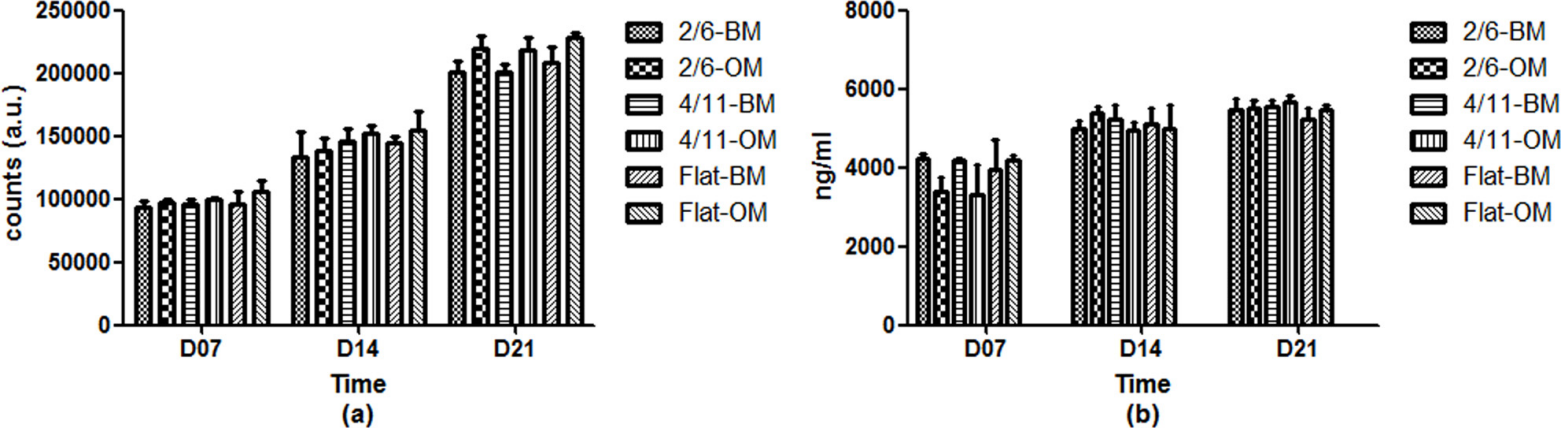
**Metabolic activity (a) and DNA amount (b) of MG-63 cells cultured on 2/6, 4/11 and flat surface in basic or osteogenic medium for up to 21 days.** A gradual increase in both metabolic activity and DNA amount was observed for all surfaces and in both media, without significant effects of surface topography.

PrestoBlue analysis of metabolic activity, performed after 7, 14 and 21 days of culture (Fig 4A) showed that all surfaces supported the growth of MG-63 cells. Over the 21-day culture period, the metabolic activity of the cells increased steadily in both BM and OM, for both microgrooved topographies and the flat control. No significant effect of the medium or the topography was observed on cell viability.

Quantification of DNA amounts (Fig 4B) was in accordance with metabolic activity data. A slow increase in DNA amount was observed on all substrates over the culture period of 21 days in both BM and OM. At 7 days, a slightly lower DNA amount was measured for cells cultured in OM on 2/6 and 4/11, however, no significant differences were observed between patterned and unpatterned PS. The cells were semi-confluent after 7 days and reached full confluence at the later time points. While it is known that confluence of cells may have a negative effect on osteogenic differentiation, the fact that no significant differences in cell proliferation were observed on different materials makes the comparison of osteogenic differentiation among them still possible.

These data suggested that microgrooves used in this experiment did not have a strong effect on cell metabolic activity or proliferation.

### Osteogenic differentiation of MG-63 cells on microgrooved PS

**Fig 5:**
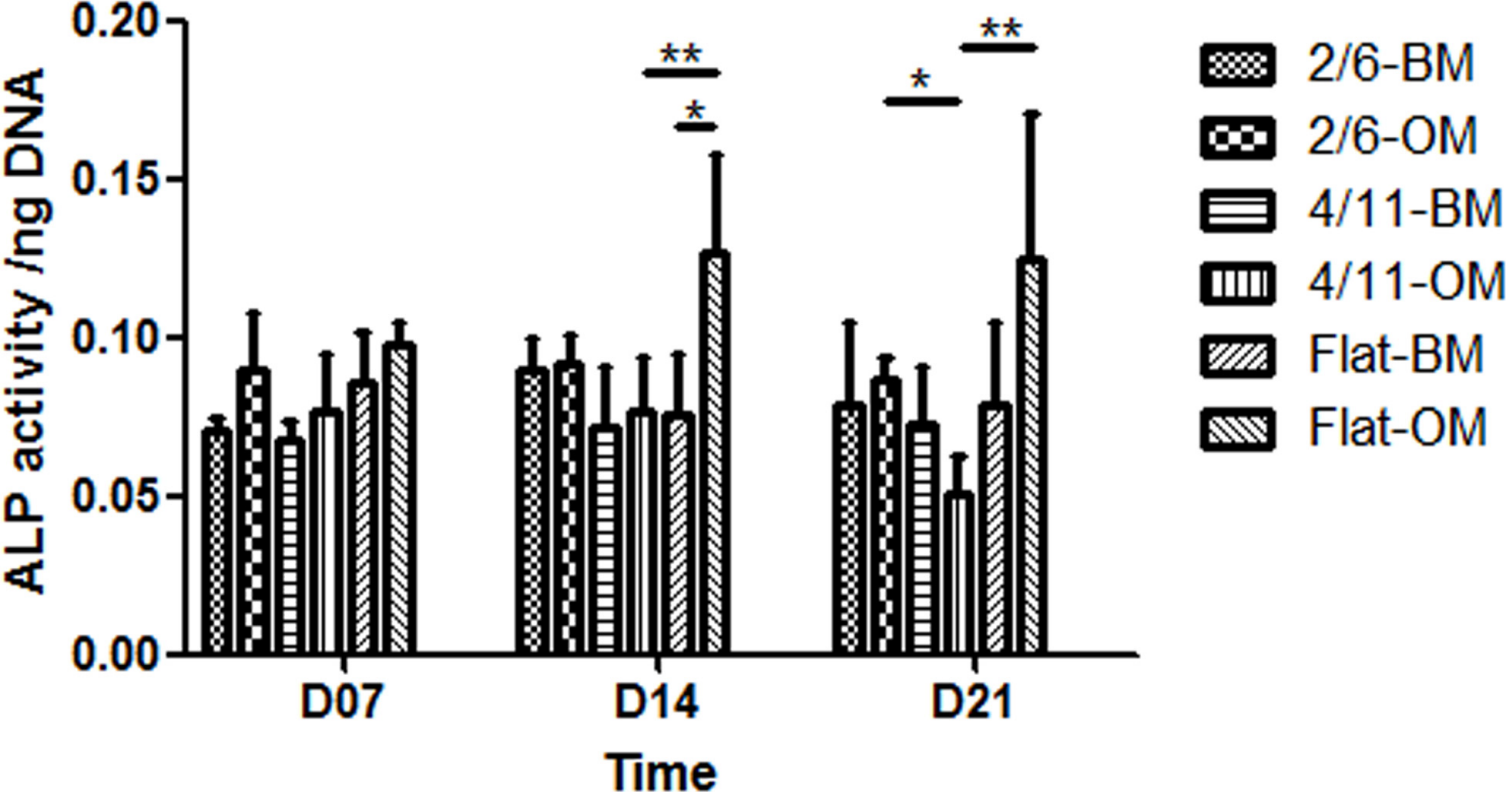
ALP activity, normalized to DNA amount of MG-63 cells cultured on 2/6, 4/11 and flat surface in basic or osteogenic medium for up to 21 days. The effect of topography on the ALP activity was mild, with cells cultured in OM on 4/11 showing a significantly lower activity as compared to the flat control at 14 days, and to both the flat control and 2/6 at 21 days. Statistically significant differences are marked with * for *p* < 0.05 and **for *p* < 0.01.

Based on the assumption that a potential effect of the initial cell morphology change caused by the surface topography on osteogenic differentiation would be observed later, 7, 14 and 21 days were selected to measure ALP activity and mRNA transcript expression. ALP enzymatic activity (Fig 5) remained at comparable levels throughout the 21-day cell culture period for all samples, with the exception of cells cultured on the unpatterned substrate in OM, the activity of which increased between day 7 and 14. In BM, no significant effect of the surface topography was observed at any time point. In OM, however, a higher ALP activity was observed on the flat PS as compared to 4/11 at day 14 and day 21. Furthermore, cells cultured on 2/6 also showed a higher ALP activity as compared to those cultured on 4/11 at 21 days.

**Fig 6:**
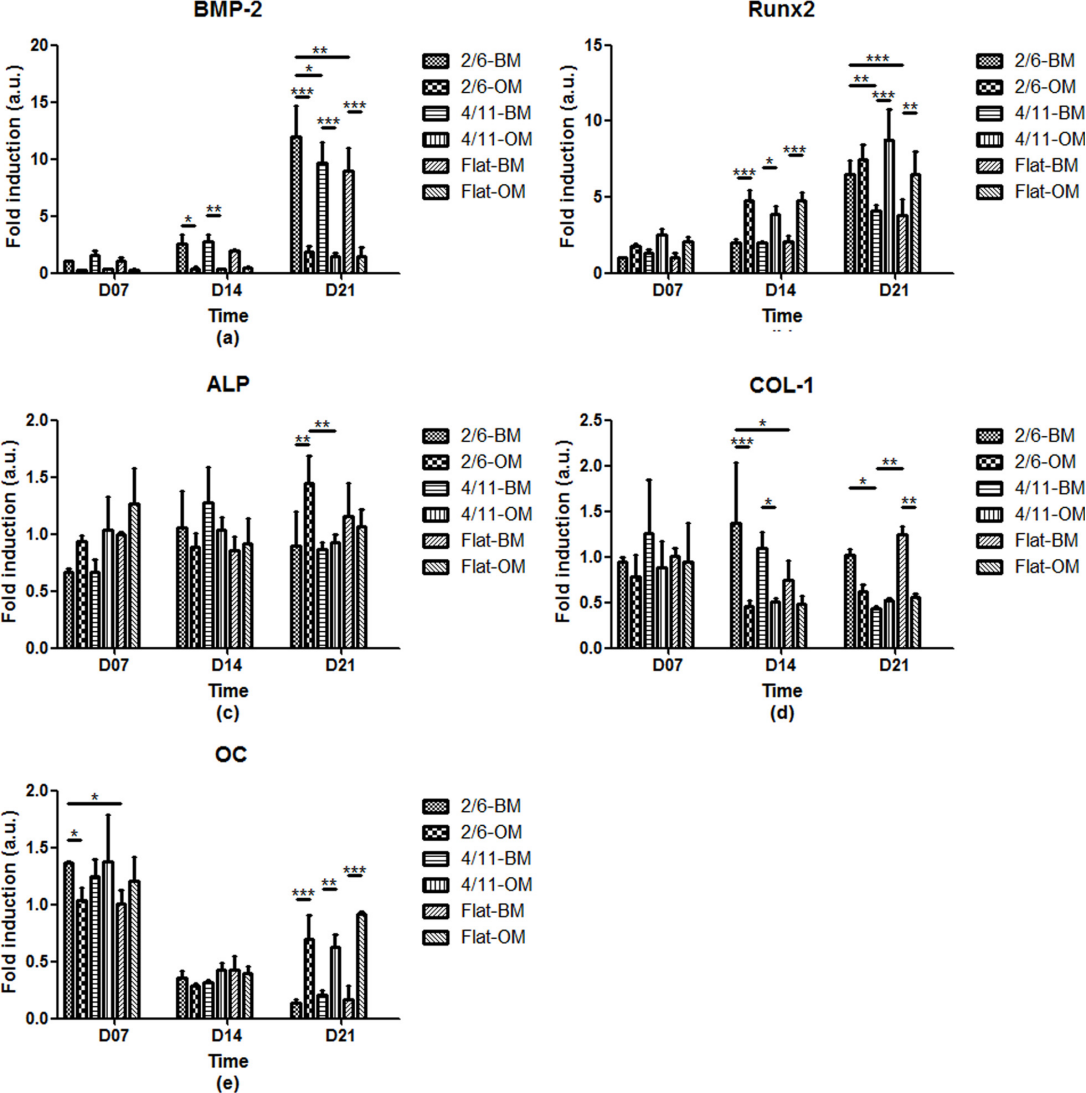
Normalized expression of a panel of osteogenic markers at mRNA level of MG-63 cells cultured on 2/6, 4/11 and flat surface in basic or osteogenic medium for up to 21 days. Cells cultured on 2/6 in BM for 21 days showed a higher expression of BMP-2 and Runx2 than the cells cultured on 4/11 or flat substrate. A similar effect was observed for ALP expression after 21 days in OM. Col-1 expression in BM by cells cultured on 2/6 was higher in comparison to the flat control after 14 days and in comparison to 4/11 after 21 days. The only topography effect on OC expression was seen after 7 days, where the cells cultured on 2/6 showed a higher expression as compared to the flat control. Statistically significant differences are marked with * for *p* < 0.05, **for *p* < 0.01 and ***for *p* < 0.001.

To further investigate the differentiation of MG-63 cells at mRNA level, the expression of a panel of osteogenic markers, BMP-2, Runx2, ALP, Col-1 and OC was investigated upon 7, 14 and 21 days of culture in BM or OM (Fig 6).

A temporal increase in the expression of BMP-2, a highly expressed marker in cells involved in bone morphogenesis [36, 37], was observed on all substrates and independent of the medium used. In general, the BMP-2 expression was higher when cells were cultured in BM as compared to OM, with significant differences for 2/6 and 4/11 at 14 days, and for all substrates at 21 days. While in OM, no significant effect of the PS surface topography was observed at any of the time points, in BM at 21 days, the BMP-2 mRNA expression of cells cultured on 2/6 was significantly higher as compared to the other two substrates.

The expression of Runx2, an osteoblast-specific transcription factor indicative of the initiation of osteogenesis [38], also increased in time, comparable to the BMP-2 expression. However, in contrast to BMP-2, Runx2 expression was in general higher when cells were cultured in OM as compared to BM, with significant differences for all substrates at 14 days, and for 4/11, and flat control at 21 days. Regarding the effect of surface topography, in BM, the cells cultured on 2/6 for 21 days showed a significantly higher Runx2 expression than those cultured on the flat substrate or 4/11, which was in accordance with the data observed for BMP-2 mRNA expression. No significant topography effects were observed in OM.

The expression of ALP, a membrane-associated protein that is expressed during the post-proliferative period of extracellular matrix maturation [39, 40], remained relatively constant in time, which was in accordance with data for ALP enzymatic activity. In general, the ALP mRNA expression was higher when cells were cultured in OM as compared to BM, with the significant difference reached for 2/6 after 21 days. While no topography effect was observed in BM at any of the time points, at day 21 in OM, the cells cultured on 2/6 showed a higher ALP mRNA expression than cells cultured on 4/11, which was in line with the data on enzymatic activity.

The expression of Col-1, a collagenous protein that is expressed during the initial period of proliferation and matrix synthesis [39], was in general low, and no temporal changes were observed. A somewhat higher expression was observed when cells were cultured in BM as compared to OM, independent of the time point or topography. At 14 days, cells cultured on 2/6 and 4/11 in OM showed a downregulation of Col-1 expression as compared to cells cultured in BM. The same effect was observed for flat PS and 2/6 after 21 days of culture. Concerning the topography effect, in BM, cells cultured on 2/6 for 14 days showed a higher expression as compared to cells cultured on the flat substrate, whereas at 21 days, both 2/6 and the flat substrate showed a higher expression than the culture on 4/11. No topography effects were observed when cells were cultured in OM.

Finally, the expression of OC, a vitamin K- and D-dependent protein that is expressed during the mineralization stage [39, 40], remained low throughout the culture period, decreasing with time, in particular in cells cultured in BM. While at the earlier time points, the effect of the medium was only observed at 7 days for 2/6, after 21 days of culture, a significantly higher OC mRNA expression was observed in OM as compared to BM, on all substrates. The only topography effect was observed in BM after 7 days, with a higher OC mRNA expression on 2/6 as compared to the flat substrate.

The analysis of the osteogenic differentiation showed that cells cultured on 2/6, the substrate with narrow grooves and ridges, in general showed the highest expression of the osteogenic markers. This effect was seen in both BM and OM, dependent on the marker analyzed.

## Discussion

The aim of the current study was to obtain deeper understanding of the effects of microgrooves on shape and orientation of osteoblast-like cells and to relate this effect to metabolic activity, proliferation and osteogenic differentiation of the cells. In the past 10 years, a number of studies have investigated the effect of microgrooved surfaces of polymers and metals on cell behavior, with emphasis on the effect on cell shape and orientation [4, 8, 11–16]. The size of the grooves and their periodicity have been suggested to play an important role in the effect of the microtopographies on cell shape [11, 14, 16, 17, 20, 41], which is plausibly related to the relationship between the groove/ridge size and the cell size. Based on the earlier studies [16, 17, 20], which showed remarkable cell guidance on surfaces having microgrooves with dimensions comparable to the cell size, here we have selected microgrooved patterns with dimensions in the range 2 to 11 μm. Indeed, qualitative analysis showed that MG-63 cells exhibited an elongated morphology aligned in the direction parallel to the grooves, which was in contrast to the unpatterned flat substrate where the cells showed a more spread, circular morphology without clear orientation. Differences were also observed between microgrooved surfaces of different size. While on 2/6, the surface with narrower grooves and ridges, cells were mainly observed on the ridges, with a bridging effect over the grooves, on the 4 /11 surfaces, the cells were predominantly found inside the grooves. The size of the wider grooves appeared sufficient to “host” the cells, and the fact that they were constrained inside the grooves resulted in a more pronounced orientation, as compared to the topography where cells were found on the ridges.

While an obvious effect of the microgrooved topography was observed on shape and orientation of MG-63 cells, there was no influence on cell proliferation or metabolic activity. Regarding the osteogenic differentiation, ALP enzymatic activity was mildly affected by the surface topography, with 2/6, the substrate with narrow grooves and ridges, showing higher values after 21 days of culture in OM that contained dexamethasone as a biological stimulator of osteogenic differentiation. A similar mild effect was observed on the expression of ALP at mRNA level. Interestingly, the strongest effect of topography was observed when cells were cultured in BM, i.e. medium without biological stimulators of osteogenesis. Cells cultured on 2/6, with narrow grooves/ridges, showed a higher expression of BMP-2 and Runx2, and to a lesser extent of Col-1 and OC. No such effect was observed in cells cultured on 4/11, the substrate with wider surface microfeatures. Taken together, these data show that there was an effect of surface topography on osteogenic differentiation, although this effect was not strong for all markers tested, which suggests that further optimization of topographical features is needed.

Earlier studies on the microgroove effects on osteoblasts proliferation, metabolic activity and osteogenic differentiation have shown varying results. For example, it has been reported that microgrooved poly(lactic acid) surfaces do not affect osteoblast proliferation while the grooves with a width of 1 and 2 μm had a positive effect on osteogenic differentiation and bone formation [12, 13]. These results are in accordance with what was observed in the current study. The 2/6 substrate favoured osteogenic differentiation, as compared to 4/11, the substrate with wider grooves and ridges, and the flat substrate. This observation indeed suggests that the effect of microgrooves on osteogenic differentiation was dependent on the size of grooves/ridges and their periodicity. On the other hand, it has been shown that microgrooved titania limits the osteoblast proliferation and is even detrimental to their differentiation towards the osteogenic lineage at short culture periods [27]. It should be mentioned that the grooves in this study were wider than what we used here, namely 12 or 40 μm, partly explaining differences observed between the two studies. It is nevertheless also plausible that, besides, the surface topography, surface chemistry plays an important role too. This is also in accordance with studies in which the effect of microgrooves was compared between PS and poly(lactic acid) [12, 13].

A significant effect was observed for the type of medium on the osteogenic differentiation. Although no consensus exists regarding the best stimulator of osteogenic differentiation of MG-63 cells, some work has been published on the use of 1,25(OH)_2_D_3_ [42]. Here, we have selected dexamethasone, based on its earlier proven stimulatory effect on ALP activity in MG-63 cells [43, 44] although there were also studies in which no effect of ALP or OC secretion was observed [45]. Furthermore, we intended to compare this data to our previous work with hMSCs, where dexamethasone is a know stimulator of osteogenic differentiation. Our results indeed showed a positive effect of the osteogenic medium on the ALP activity, on flat controls only, whereas the effect on ALP mRNA expression was not as obvious. Furthermore, the expression of Runx2 and OC was enhanced both on microgrooved substrates and the control. The fact that BMP-2 was downregulated in presence of dexamethasone is possibly related to the fact that dexamethasone may decrease intracellular calcium levels [45], resulting in lower expression of BMP-2, which is a calcium-responsive gene. This is also in accordance with our previous work showing lower BMP-2 mRNA expression in osteogenic medium, when cultured on calcium phosphates (e.g. [46]).

Regarding the correlation between the effect of the microgrooved topography on cell shape and cell behavior in terms of osteogenic differentiation, the results of this study suggested that the cells with larger area and perimeter exhibited a more pronounced osteogenic differentiation. This corroborates with the results from previous studies on the relationship between cell spreading on fibronectin islands and osteogenic differentiation [47, 48]. It has been shown that cells cultured on larger islands of fibronectin commit to osteogenic lineage, whereas those cultured on smaller islands tend to differentiation towards the adipogenic lineage [48]. This effect may be related to enhanced contractility with increased cell spreading, that in turn promotes osteogenic differentiation [49]. Regarding cell orientation, the wider microgrooves that accommodated cell spreading and had a strongest effect on OA did not appear to positively affect the osteogenic differentiation of MG-63 cells, which is in agreement with previous results [27].

Previous studies mainly focused on the effect of microgrooved surfaces either on cell shape, orientation or on proliferation and osteogenic differentiation by detecting ALP activity or mineralized extracellular matrix production. Here, we elucidated on the effect of microgrooves/microridges on cell behaviour comprehensively from cell shape and orientation, through metabolic activity and growth, to ALP activity and expression of a panel of osteogenic markers at gene level. These data could be useful as input for developing PS-based cell culture platforms that are microstructured in such a way that they can directly affect cell behavior, in absence of biological stimulators. Finally, it should be emphasized that this study, like many others focusing on surface topography, was performed in a 2D environment, which is not a natural microenvironment for the cell. Predictive value of such a study is therefore limited for the cell behavior in 3D environment [50–53]. The challenge therefore lies is developing methods that would allow micropatterning of functional, 3D biomaterials that can be applied clinically.

## Conclusions

The results of this study have demonstrated the effect of microgrooved PS surfaces on the morphology, metabolic activity, proliferation and osteogenic differentiation of MG-63 osteoblast-like cells. The cells grown on PS surfaces with 4 μm-grooves/11 μm-ridges showed a more pronounced cell alignment and a somewhat lower cell areas and cell perimeters as compared to cells cultured on 2 μm-groove/6 μm-ridge surfaces or unpatterned PS. While no effects were observed on metabolic activity or proliferation of cells, their differentiation towards the osteogenic lineage was enhanced on the substrate with the narrower micropattern. This positive effect may be related to a more pronounced cell spreading. This study will contribute to the existing knowledge on employing surface microtopography to control cell and tissue response to biomaterials.
